# Cosmc transfection decreases malignant behavior of Tn^+^ cells and enhances sensitivity to apoptosis when induced by Apo2L/TRAIL via alteration of O-glycan structure

**DOI:** 10.18632/aging.203633

**Published:** 2021-10-13

**Authors:** Ruisong Ding, Xingyou Hu, Wen Hu, Zhenzhen Du, Panpan Huang, Mengyang Wang, Jiaoyue Sheng, Yanchao Ma, Ailing Wang, Xiying Luan, Menghua Dong, Qizhi Cao, Yanfen Zou, Tao Hu

**Affiliations:** 1 Department of Immunology, Binzhou Medical University, Yantai 264003, PR China; 2 Qingdao University, Qingdao 266071, PR China; 3 Laboratory Department, Yantai Affiliated Hospital of Binzhou Medical University, Yantai, Shandong Province 264100, PR China; 4 Department of Oncology, Qingdao No.6 People’s Hospital, Qingdao 266033, PR China; 5 Department of Obstetrics and Gynecology, The Affiliated Yantai Yuhuangding Hospital of Qingdao University, Yantai 264000, PR China

**Keywords:** Cosmc, Apo2L/TRAIL, O-glycosylation, C3GnT, Tn antigen, apoptosis

## Abstract

Cosmc mutations may cause abnormal O-glycosylation and result in Tn antigen expression. In the current study, it was discovered that proliferation and migration of Tn+ cells (Jurkat T and LS174T-Tn^+^ cells) with mutant Cosmc decreased after transfected Cosmc, and their sensitivity to apoptosis induced by Apo2L/TRAIL increased. Core 1-, 2-, and 3-derived O-glycans were absent in Tn^+^ cells. After Cosmc transfection, normal extended core 1-derived O-glycans appeared and were accompanied by increased T-synthase activity. Core 2-derived O-glycans appeared in transfected LS174T-Tn^+^ cells, and their structural types and levels were lower than those in LS174T-Tn^−^ cells. Core 3-derived O-glycans were present only in LS174T-Tn^−^ cells. The activity of C3GnT in LS174T-Tn^+^ cells was lower than that in LS174T-Tn^−^ cells, and it was absent in Jurkat T cells. Cosmc transfection did not alter C3GnT activity or core 3-derived O-glycans in Jurkat T and LS174T-Tn^+^ cells. The results demonstrated that the composition and structure of O-glycans were different among various Tn^+^ cells, which not only affected cell malignant behavior but also modulated sensitivity to apoptotic stimuli. Thus, Cosmc transfection may effectively decrease the malignant behavior of Tn^+^ tumor cells and enhance their sensitivity to apoptosis when induced by Apo2L/TRAIL through modification of O-glycans.

## INTRODUCTION

Colorectal cancer (CRC) is the third most common malignant tumor. Although the morbidity and mortality of CRC have shown downward trends among the elderly, the morbidity of CRC has remained high among young adults [1]. In order to clarify the pathogenesis of CRC, the effects of glycosylation on tumorigenesis have received increased attention, especially abnormal O-glycosylation resulting in the truncation of immature O-glycans Tn antigen (GalNAc-Ser/Thr) expressed on cell surfaces. This is thought to be related to the malignant transformation of normal human cells into CRC cells [2, 3]. Moreover, with the application of the cellular O-glycome reporter/amplification (CORA) method, exploring the effects of O-glycan modification on glycoprotein and cellular glycomics and how this correlates with biological function has become easier and more precise [4]. This is helpful to further clarify the correlation between O-glycosylation and tumor occurrence and development.

Normally, T-synthase transfers galactose (gal) from a donor (UDP-gal) to GalNAc (Tn antigen) to form the core 1 structure (gal[β1-3] GalNAc-Ser/Thr, T antigen), which can be further sialylated and form a ST antigen via sialyltransferase ST3Gal-I activity in all cell types. Core 3 β1-3 N-acetylglucosaminyltransferase (C3GnT) also transfers GlcNAc from a donor (UDP-glcNAC) to GalNAc to form the core 3 structure in gastrointestinal epithelia, which is further modified to form an extended complex core 3 structure that includes sialyl-Le^X^ or sulfo-Le^X^ on core 3 O-glycans [5]. The Tn antigen is a common tumor-associated carbohydrate antigen barely expressed in normal tissues, but it is expressed on the surfaces of many tumor cells (i.e., in gastric, colorectal, lung, ovarian, and breast cancers) [6–10]. The molecular mechanisms of Tn antigen expression are thought to be related to abnormalities in the molecular chaperone Core1β3 galactosyltransferase (T-synthase)-specific molecular chaperone (Cosmc). Decreased levels or loss of T-synthase activity resulting from Cosmc mutations eventually leads to abnormal O-glycosylation and expression of Tn antigen [11]. Moreover, knockdown of Cosmc may promote oncogenesis [12], since the high expression of Tn and sialylated Tn antigen (STn) is closely related to the clinical stage and prognosis in various tumor tissues [13].

Alterations in glycosylation patterns have been shown to be associated with cell apoptosis. O-glycosylation is critical for the stability of glucose-regulated protein 78 (GRP78) that participates in endoplasmic reticulum (ER) stress, autophagy, and inhibited tumor cell apoptosis [14]. As a potential target drug for effective cancer treatment, Apo2L/TNF-related apoptosis-inducing ligand (Apo2L/TRAIL), a cytokine in the TNF superfamily, has the ability to selectively induce tumor apoptosis [15]. It can also selectively kill cancer cells and cause depolarization, regulate the levels of reactive oxygen species (ROS), and interfere with mitochondrial and ER function [16]. Its receptors, also called death receptors (DR4 and DR5), are membrane glycoproteins containing O-glycosylation sites. They combine with Apo2L/TRAIL, resulting in the aggregation of Fas-related death domains and caspase 8, and they also initiate cell apoptosis [17]. Related reports have confirmed that the O-glycosylation status of DR4 and DR5 alters the structure of DR4/5 on the surfaces of tumor cells through GalNAc transferase (GALNT14), thereby changing Apo2L/TRAIL-induced apoptosis [18]. Moreover, DR4 receptor-specific ligands are superior to DR5 ligands in triggering cancer apoptosis signals, and downregulation of DR4 leads to a decrease in the susceptibility of colorectal cancer cells to Apo2L/TRAIL [19]. So far, however, it is unclear whether changes in O-glycans result from abnormal Cosmc with biological behavior of tumor cells and whether this is caused by the sensitivity to apoptosis induced by Apo2L/TRAIL.

In this study, T-synthase activity was detected using a previously described fluorescence method and C3GnT activity was detected using an original method innovated for this study. Changes in O-glycans were detected using cellular O-glycome reporter/amplification (CORA). Comparisons were made among proliferation, migration, and apoptosis induced by Apo2L/TRAIL in Jurkat T cells and LS174T-Tn^+^ cells isolated from CRC cell line LS174T that were untransfected and transfected with WtCosmc Tn^+^ cells harboring mutant Cosmc. It was found that transfected WtCosmc influenced the activity of T-synthase and core 1- and core 2-derived O-glycans, proliferation and migration of Tn^+^ tumor cells, and apoptosis induced by Apo2L/TRAIL; however, it did not change the activity of C3GnT and core 3-derived O-glycans.

## RESULTS

### Tn^+^ and Tn^−^ cells coexisted in human CRC cell lines LS174T and transfected wild-type Cosmc inhibited Tn antigen expression

**Figure 1:**
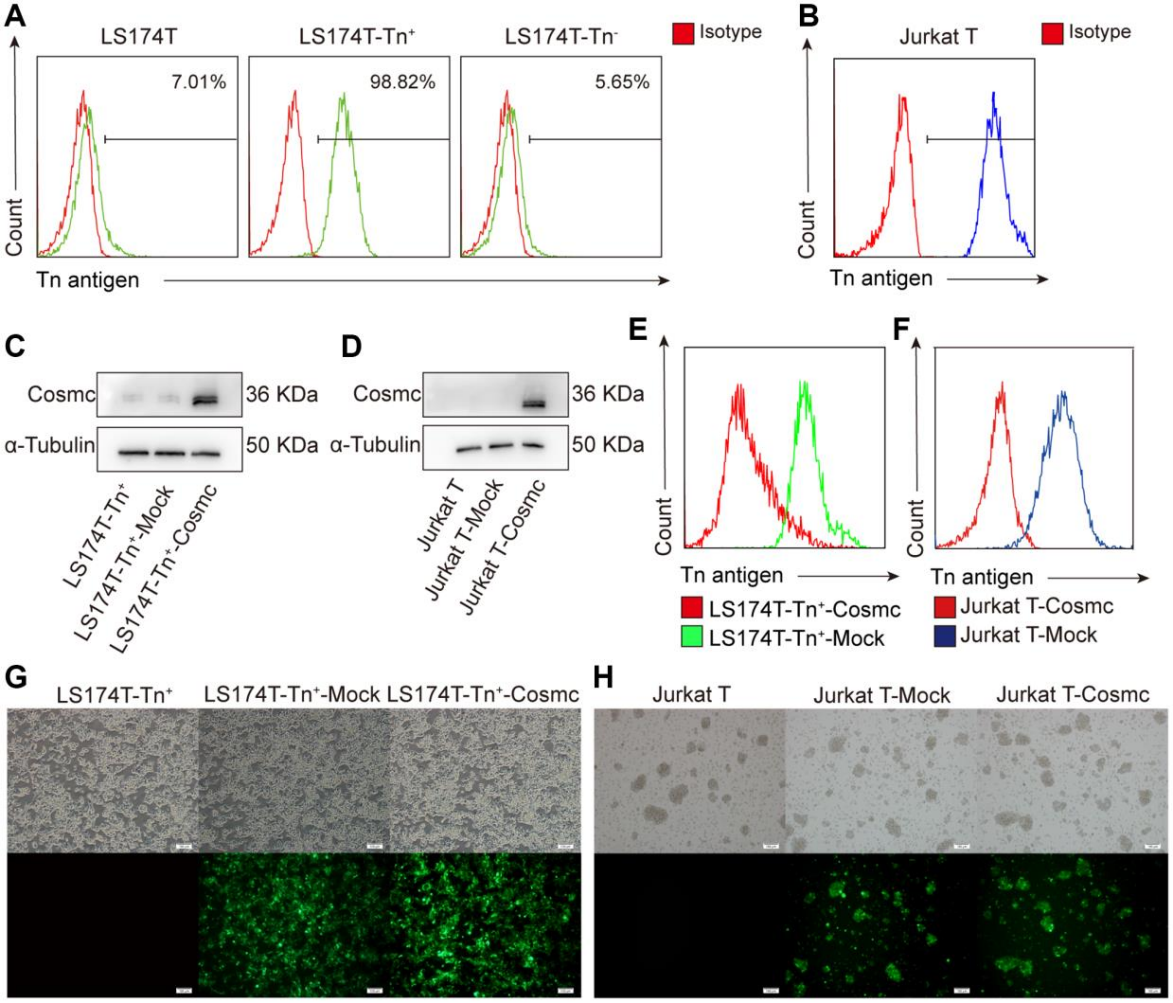
**Transfection WtCosmc in LS174T cells and Jurkat T cells.** (**A**) The percentage of Tn^+^ cells in LS174T cells and sorted LS174T-Tn+and Tn^−^ cells by magnetic bead separation. (**B**) The percentage of Tn^+^ cells in Jurkat T cells. (**C**) Western blot analysis of Cosmc expression in LS174T-Tn^+^, LS174T-Tn^+^-Mock, and LS174T-Tn^+^-Cosmc cells. (**D**) Cosmc expression in Jurkat T, Jurkat T-Mock, and Jurkat T^−^Cosmc cells. (**E**) FCM analysis of the percentage of Tn^+^ cells in LS174T-Tn^+^-Mock and LS174T-Tn^+^-Cosmc cells. (**F**) The percentage of Tn^+^ Cells in Jurkat T-Mock and Jurkat T-Cosmc cells. (**G**) WtCosmc and Mock transfection cells with green fluorescence protein in LS174T-Tn^+^ cells as detected by immunofluorescence microscopy. (**H**) WtCosmc and Mock transfection cells with green fluorescence in Jurkat T cells (scale bars = 100 μm).

Previous studies have shown that Cosmc mutation decreases the activity of T-synthase and results in Tn antigen expression. According to the expression of Tn antigen on the cell surface, tumor cells were divided into Tn^+^ and Tn^−^ cells. Using flow cytometry, it was found that there were approximately 7.01% Tn^+^ cells in human CRC cell line LS174T (Figure 1A) and almost 100% Tn^+^ cells in Jurkat T cells (Figure 1B). The Tn^+^ cells have been confirmed to exist in mutant Cosmc cells [20, 21] and in the absence of Cosmc protein.

After stably transfecting a plasmid encoding the *WtCosmc* gene, Cosmc protein levels increased, and the percentage of Tn^+^ cells decreased significantly (Figure 1C–1F). There was a small amount of Cosmc protein in LS174T-Tn^+^ cells before and after transfection with Mock plasmid. This is potentially due to the separation of LS174T-Tn^+^ cells from LS174T containing residual Tn^−^ cells. Although transfection efficiency is not 100%, the successfully transfected cells possessed green fluorescent labels (EGFP) and no longer expressed Tn antigen (Figure 1G and 1H).

### Tn^+^ cells showed a stronger ability to proliferate and migrate than corresponding Tn^−^ cells, which was downgraded by WtCosmc transfection

**Figure 2:**
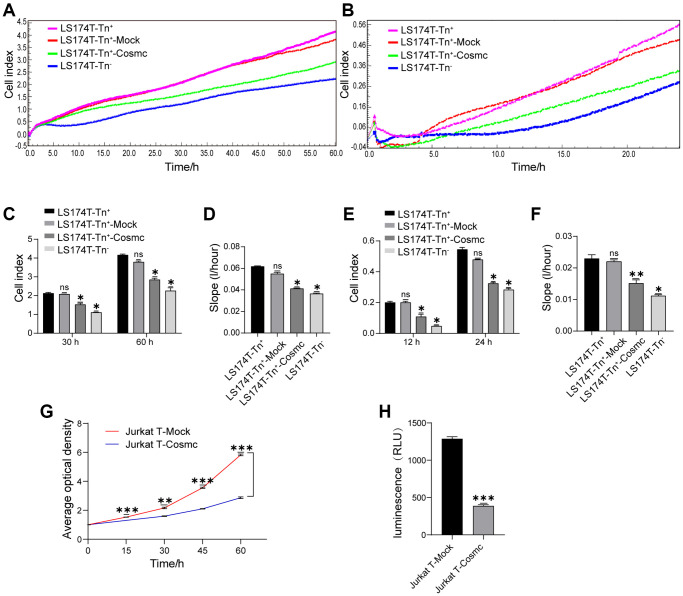
**Cosmc transfection downregulated Tn^+^ cell proliferation and migration.** (**A**–**F**) Proliferation and migration in LS174T-Tn^−^ and LS174T-Tn^+^ cells before and after transfection with WtCosmc or Mock were detected by RTCA. (**A**) Proliferation curve, (**B**) Migration curve, (**C**) Cell index of proliferation at typical timepoints (30 h and 60 h), (**D**) Slopes for proliferation at 0–60 h, (**E**) Cell index of migration at typical timepoints (12 h and 24 h), and (**F**) slopes for migration at 0–24 h. (**G**) The proliferation ability of Jurkat T cells transfected with Cosmc or Mock was detected by CCK-8 assay at different timepoints (15 h, 30 h, 45 h, and 60 h). (**H**) The migration of Jurkat T cells transfected with Cosmc or Mock was detected using a transwell assay for 24 h. Data shown are the mean ± SD of three independent experiments (^*^P < 0.05, ^**^P < 0.01, ^***^P < 0.001).

The Tn antigen, as an O-glycoprotein with truncated O-glycans expressed on its cell surface, can influence cell-cell interactions and affect the biological behavior of Tn^+^ cells. The RTCA showed that proliferation and migration of LS174T-Tn^+^cells were higher than those of corresponding Tn^−^ cells. After transfection with WtCosmc, the proliferation and migration of LS174T-Tn^+^ cells declined significantly (Figure 2A–2F). Since Jurkat T cells were non-adherent and could not be analyzed by RTCA, their proliferation and migration were evaluated by CCK8 and transwell assays, respectively. The results showed that the ability of Jurkat T cell proliferation and migration also decreased after transfection with WtCosmc (Figure 2G and 2H).

### Sensitivity to TRAIL-induced apoptosis for Jurkat T cells and LS174T-Tn^+^ cells was enhanced after transfection with WtCosmc

**Figure 3:**
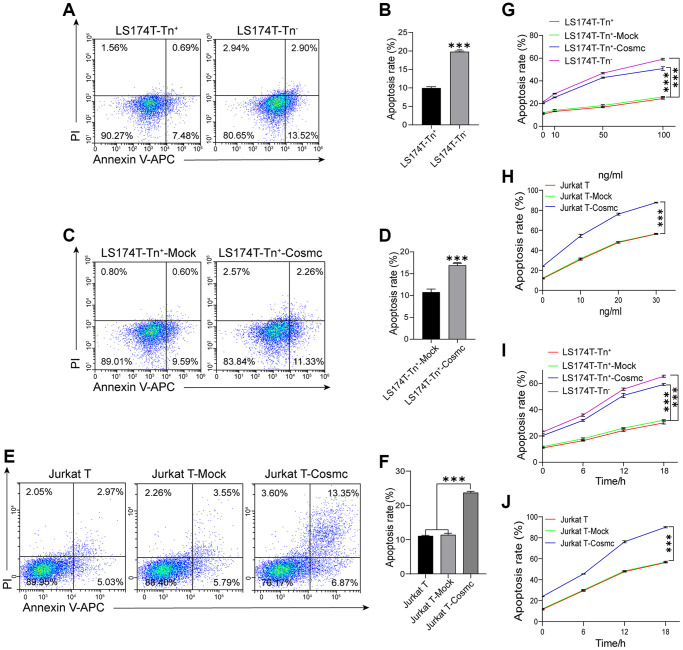
**Apoptosis of Tn^+^ cells analysis with FCM before and after treatment with WtCosmc and Apo2L/TRAIL.** (**A**–**B**) Apoptosis rates in LS174T-Tn^+^ and LS174T-Tn^−^ cells. (**C**–**D**) Apoptosis rates in LS174T-Tn^+^-Mock and LS174T-Tn^+^-Cosmc cells. (**E**–**F**) Apoptosis rates in Jurkat T, Jurkat T-Mock, and Jurkat T-Cosmc cells. (**G**) Apoptosis of LS174T-Tn^−^ and LS174T-Tn^+^ cells after exposure to Apo2L/TRAIL at different concentrations (10 ng/mL, 50 ng/mL, and 100 ng/mL) before and after transfection with WtCosmc or Mock. (**H**) Apoptosis of Jurkat T cells after exposure to Apo2L/TRAIL at different concentrations (10 ng/mL, 20 ng/mL, and 30 ng/mL) before and after transfection with WtCosmc or Mock. (**I**) The apoptosis of LS174T-Tn^−^ and LS174T-Tn^+^ cells were exposed to Apo2L/TRAIL at 100 ng/mL and different timepoints (6 h, 12 h, and 18 h) before and after transfection with WtCosmc or Mock. (**J**) Apoptosis of Jurkat T cells after exposure to Apo2L/TRAIL at 20 ng/mL and different timepoints (6 h, 12 h, and 18 h) before and after transfection with WtCosmc or Mock. Data shown are the mean {plus minus} SD of three independent experiments (^***^P < 0.001).

Loss of T-synthase activity in tumor cells results in the expression of Tn antigen. Cosmc promoter methylation has been shown to decrease the levels of Cosmc protein and increase the expression of Tn and STn antigens in breast cancer. Reduced methylation can inhibit cell growth, migration, and invasion, promote apoptosis in breast cancer cells *in vitro,* and restrain tumor growth *in vivo* [22]. Our results showed that apoptotic rates of LS174T-Tn^+^ cells were lower in LS174T-Tn^−^ cells (Figure 3A and 3B) and increased after transfection with WtCosmc. Similarly, Jurkat T cells with lower apoptosis rates also increased after WtCosmc transfection; however, apoptosis rates in Tn^+^ cells before and after transfection with Mock plasmid showed no significant changes (Figure 3C–3F). These changes may indicate that normal Cosmc activity contributes to cell apoptosis.

In addition, research has confirmed that the O-glycosylation degree of DR4 and DR5 alters Apo2L/TRAIL-induced apoptosis [18]. Thereby, the increased apoptosis in transfected WtCosmc cells was induced by Apo2L/TRAIL, which suggests that Cosmc transfection may alter the homo-oligomerization of DR4 and DR5. Interestingly, Apo2L/TRAIL may induce apoptosis of Jurkat T, Jurkat T-Mock, Jurkat T-Cosmc, LS174T-Tn^+^, LS174T-Tn^+^-Mock, LS174T-Tn^+^-Cosmc, and LS174T-Tn^−^ cells in a dose-dependent manner (Figure 3G and 3H).

By contrast, apoptosis rates in Jurkat T-Cosmc cells and LS174T-Tn^+^-Cosmc cells were higher than those in Jurkat T-Mock cells and LS174T-Tn^+^-Mock cells. Apoptosis rates in both Tn^+^ cells before and after transfection with Mock plasmid showed no significant changes. Meanwhile, apoptosis rates of LS174T-Tn^+^, LS174T-Tn^+^-Mock, and LS174T-Tn^+^-Cosmc cells in 100 ng/mL Apo2L/TRAIL (in addition to Jurkat T, Jurkat T-Mock, and Jurkat T-Cosmc cells in 20 ng/mL Apo2L/TRAIL) increased with extensions in treatment time (6 h, 12 h, and 18 h; Figure 3I and 3J). These results suggest that the overexpression of Cosmc may alter the homo-oligomerization of DR4/DR5 and increase the sensitivity of Tn^+^ cells to apoptosis when induced by Apo2L/TRAIL.

### WtCosmc transfection increases T-synthase activity and extends core 1- and core 2-derived O-glycans in Tn^+^ cells

**Figure 4:**
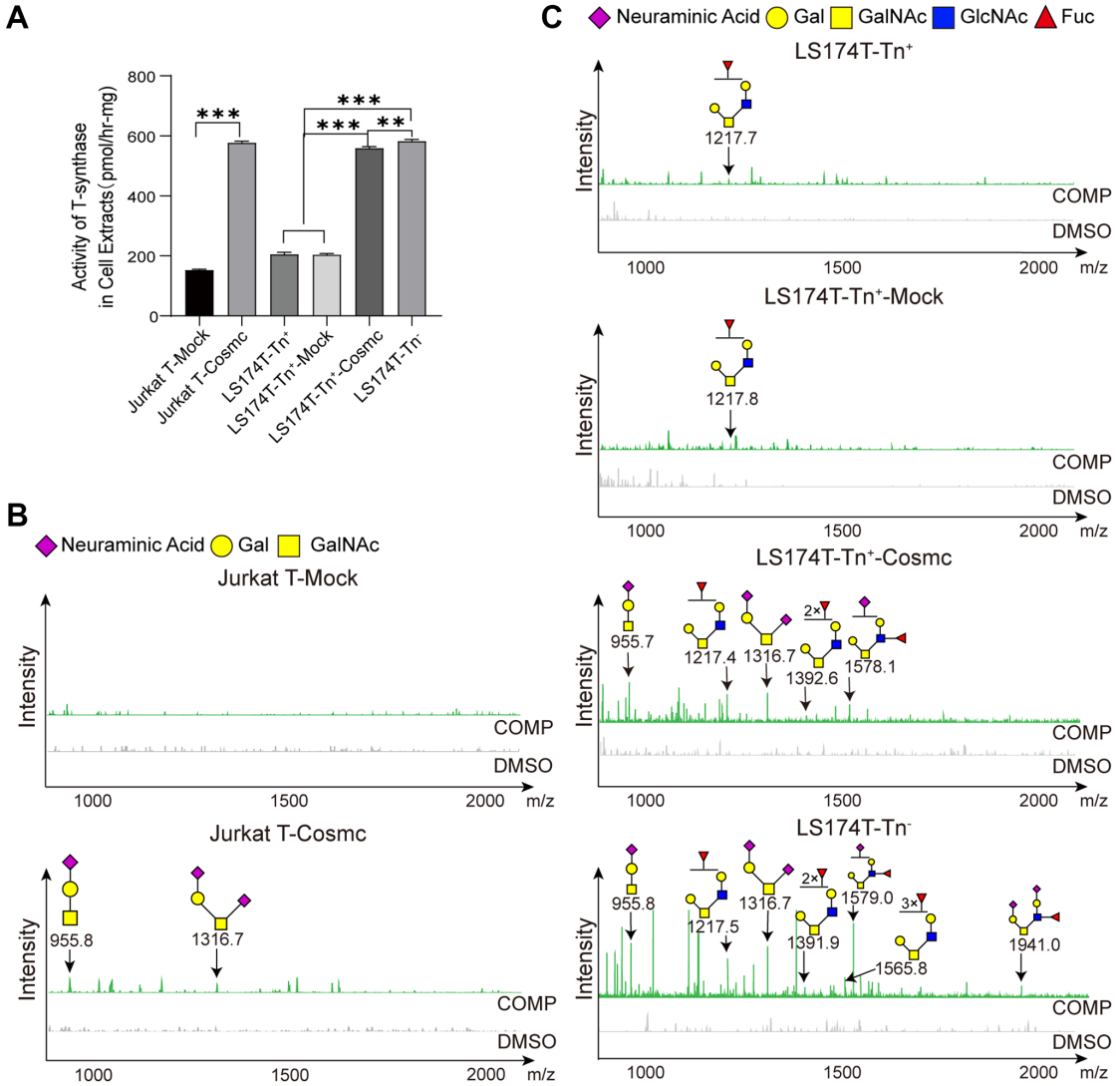
**Cosmc transfection increased the activity of T-synthase and changed core 1^−^ and core 2-derived O-glycans in Tn^+^ cells.** (**A**) The activity of T-synthase was detected using a fluorescence method in LS174T-Tn^−^, LS174T-Tn^+^, and Jurkat T cells before and after transfection with WtCosmc or Mock. (**B**) Core 1- and core 2-derived O-glycans in Jurkat T cells before and after transfection with WtCosmc or Mock were analyzed using CORA and MALDI-TOF-MS. (**C**) Core 1^−^ and core 2-derived O-glycans in LS174T-Tn^−^ and LS174T-Tn^+^ cells before and after transfection with WtCosmc or Mock (^**^P < 0.01, ^***^P < 0.001).

Cosmc is a molecular chaperone of T-synthase and is necessary for normal T-synthase activity, which participates in the correct extension of core 1-derived O-glycans. After transfection with WtCosmc, the activity of T-synthase was enhanced obviously (Figure 4A). Additionally, core 1-derived O-glycans (m/z: 955.4, 1316.6) appeared in Jurkat-T-Cosmc and LS174T-Tn^+^-Cosmc cells. Core 2-derived O-glycans (m/z: 1217.4, 1392.6, and 1578.1) based on core 1 structure were presented in LS174T-Tn^+^-Cosmc cells rather than in Jurkat T-Cosmc cells. This indicates that Jurkat T cells may have lower or lack of C2GnT activity.

Compared to LS174T-Tn^−^ cells, levels of core 2-derived O-glycans (m/z: 1217.6, 1391.6, and 1578.7) in LS174T-Tn^+^-Cosmc cells were lower, and two types of O-glycans (m/z: 1565.7 and 1940.0) were absent (Figure 4B and 4C). These differences may perhaps be due to core 1-derived O-glycans resulting from Cosmc transfection or glycosyltransferase (C2GnT, fucosyltransferase and α-2,3 sialyltransferase) the activity was insufficient in LS174T-Tn^+^-Cosmc cells. It should be noted that there were low levels of core 2-derived O-glycans (m/z: 1217.6) in LS174T-Tn^+^ and LS174T-Tn^+^-Mock cells, which may be related to a few residual Tn^−^ cells.

Importantly, normal O-glycosylation promoted ligand-stimulated clustering of DR4 and DR5, which affected Apo2L/TRAIL-induced apoptosis in tumor cells [18]. Therefore, the increased sensitivity to apoptosis induced by Apo2L/TRAIL in Cosmc-transfected cells may be correlated with proper extension of O-glycans on the cell surface.

### Cosmc transfection did not change activity of C3GnT and core 3-derived O-glycans

**Figure 5:**
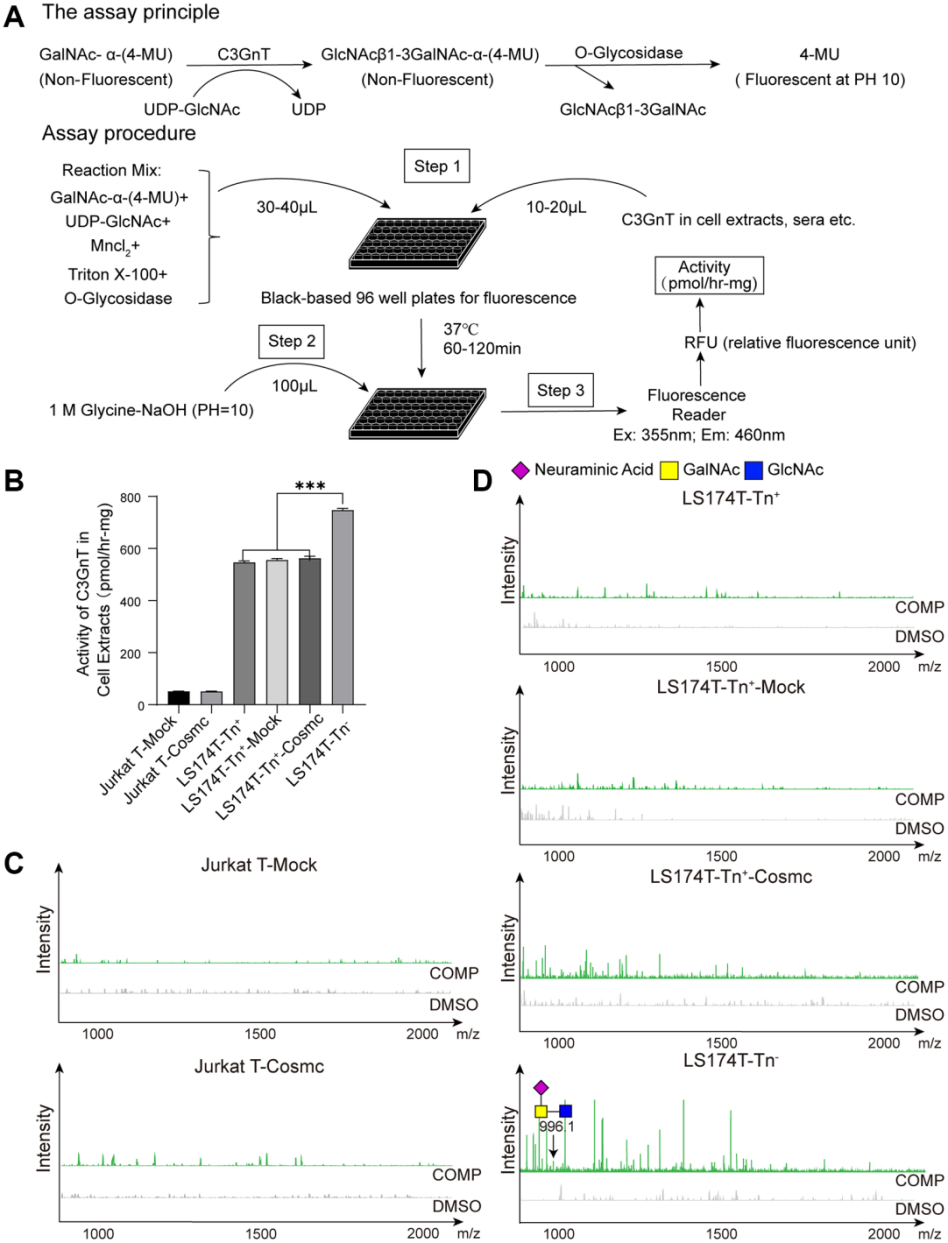
**Cosmc transfection did not influence the activity of C3GnT and core 3-derived O-glycans in Tn^+^ cells.** (**A**) The procedure of fluorescence detection of C3GnT activity. (**B**) The activity of C3GnT was detected using a fluorescence method in LS174T-Tn^−^, LS174T-Tn^+^, and Jurkat T cells before and after transfection with WtCosmc or Mock. (**C**) Core 3-derived O-glycans in Jurkat T cells before and after transfection with WtCosmc or Mock were analyzed using CORA and MALDI-TOF-MS. (**D**) Core 3-derived O-glycans in LS174T-Tn^−^ and LS174T-Tn^+^ cells before and after transfection with WtCosmc or Mock (^***^P < 0.001).

Extension of O-glycans is a complex process and requires the participation of glycosyl-transferase. The formation of core 1 structure requires T-synthase and Tn as substrates, and core 2 structure requires C2GnT and core 1-derived O-glycans as substrates. The formation of core 3 structure requires C3GnT, which mainly exists in gastrointestinal epithelia, and Tn as substrates. Using the new method presented here (Figure 5A), it was found that the activity of C3GnT in Jurkat T cells was slightly lower in LS174T-Tn^+^ cells than in LS174T-Tn^−^ cells, which did not significantly change after transfection with WtCosmc (Figure 5B). Core 1-derived O-glycans (m/z: 955.4, 1316.6) appeared in Jurkat-T-Cosmc, LS174T-Tn^+^-Cosmc, and LS174T-Tn^−^ cells. In contrast, core 3-derived O-glycans (m/z: 996.4) were only present in LS174T-Tn^−^ cells (Figure 5C and 5D).

## DISCUSSION

Protein O-glycans play important physiological functions in the formation of blood vessels and lymphatic vessels, cell adhesion, and information transmission [23–25]. O-glycosylation not only influences malignant transformation of endothelial cells [26] but also affects tumor cell characteristics that correlate with tumor-associated sugar antigens (i.e., Tn and STn antigens). In gastrointestinal tumors, the expression of STn and Tn antigens is regarded as a marker of poorly differentiated adenocarcinoma and mucinous carcinoma, and it is related to tumor aggressiveness, high proliferation, metastasis, and poor clinical prognosis [27, 28]. Tumor cells expressing STn antigen can reduce adhesion, enhance migration ability, and promote metastasis and malignancy [29]. Mucin antigens with Tn and STn epitopes are often expressed in advanced tumor stages. The presented results indicate that Jurkat T and LS174T-Tn^+^ cells express Tn antigens and have strong proliferation and migration abilities (Figure 2). The reason for these conclusions is that their O-glycans are very simple and show an absence of extension in core 1-derived O-glycans.

The other important role of O-glycosylation is regulation of cell growth through apoptosis pathways. Polypeptide N-acetylgalactosaminyltransferases 18 (ppGalNAc-T18) silencing in cells decrease O-glycosylation levels and activate ER stress, leading to apoptosis [30]. Moreover, T-synthase is a key enzyme catalyzing the formation of T antigen and ensures the normal progress of O-glycosylation [31, 32]. The decrease or deletion of T-synthase activity resulting from Cosmc mutation caused abnormal O-glycosylation, which led to Tn and STn antigen expression. Thus, WtCosmc plasmid transfection was able to restore T-synthase activity and inhibit the expression of Tn antigen (Figure 1E and 1F, Figure 4A).

The results also showed that apoptotic rates of LS174T-Tn^+^ cells (with high levels of proliferation and migration abilities) were lower than LS174T-Tn^−^ cells (Figure 3A and 3B). By contrast, Cosmc transfection reduced the proliferation and migration ability of Tn^+^ cells and promoted apoptosis (Figure 2, Figure 3C–3F). This demonstrated that high levels of proliferation and migration were potentially correlated with low apoptosis activity. The truncated O-glycans (especially Tn antigens on the cell surface) also contributed to cell proliferation, migration, and anti-apoptosis activity. Complete glycosylation and normal O-glycans extension through transfection with Cosmc may inhibit certain malignant behaviors of tumor cells.

Regarding apoptosis, the structure of receptor and its ability to combine with corresponding ligands play a decisive role. In recent years, Apo2L/TRAIL has become a research focus due to its apoptosis-inducing effects on tumor cells and lack of toxicity to normal cells. After Apo2L/TRAIL binds to the extracellular domain of death receptors (DRs), the intrinsic apoptosis signal pathway is initiated, which causes the activation of caspase 8 through the death-inducing signaling complex (DISC) [33]. The activated caspase 8 further induces apoptosis in two manners. First, it can activate caspases-3, -6, and -7, eventually resulting in apoptosis. By contrast, it also can cleave Bcl-2 interacting domain (Bid) into truncated Bid (tBid), which then triggers the intrinsic apoptotic pathway by activation of caspase-9 and caspase-3 [34, 35].

Due to the fact that receptors DR4 and DR5 contain O-glycosylation sites, the glycosylation status and level of DR4/DR5 have effects on apoptosis. The presented results showed that WtCosmc transfection effectively enhanced the sensitivity of Tn^+^ cells to apoptosis when induced by Apo2L/TRAIL in time- and dose-dependent manners (Figure 3G–3J). It was speculated that incomplete glycosylation could cause the extracellular domain of DR4 and DR5 to not effectively bind with Apo2L/TRAIL. The results of CORA showed that the O-glycan expression profiles and structures were different in Tn^+^ cells before and after treatment with WtCosmc and Tn^−^ cells. Additionally, Jurkat T-Cosmc, LS174T-Tn^+^-Cosmc, and LS174T-Tn^−^ cells expressed the core 1-derived O-glycan (m/z: 955.4 and 1316.6). Specifically, the structure of core 1 O-glycan (m/z: 1316.6) is sialyl gal-galNAc (i.e., ST antigen on the cell membrane surface may be helpful to apoptosis). This is consistent with the finding that DR4/5 carrying the ST antigen is more sensitive to Apo2L/TRAIL than cells with Tn/STn antigens [21]. Moreover, the inhibitor of glycosylation (benzyl-α-GalNAc) blocked oridonin plus TRAIL-induced apoptosis. Additionally, there are some core 2-derived O-glycan structures with glcNAc in LS174T-Tn^+^-Cosmc (m/z: 1217.6, 1391.6, and 1578.7) and LS174T-Tn^−^ cells (m/z: 1217.6, 1391.2, 1565.7, 1578.7, and 1940.0). GlcNAc promotes the clustering and activation of DR5 by improving O-glycosylation, thereby enhancing the apoptosis induced by TRAIL and recruiting the protease caspase-8, which plays a role in the initiation of apoptosis [36].

In fact, Apo2L/TRAIL contains different receptors, and many receptors are decoy receptors, such as DcR2. The expression of Tn and STn antigens can prevent the homo-oligomerization of membrane glycoproteins DR4/DR5. They can also promote hetero-oligomerization between DR5 and DcR2 lacking the death domain, thereby reducing the death signal of DR5 [21]. Studies have shown that Cosmc transfection in Jurkat T and LS174T-Tn^+^ cells do not affect the expression levels of DR4 and DR5, but could promote homo-oligomerization of DR4/DR5 by the extended sialyl core 1 O-glycans. The normal O-glycosylated DR5 in Jurkat T-Cosmc cells was more stable than the DR5 carrying Tn antigen [21], and the complex O-glycosylation of DR4 in LS174T-Tn^+^-Cosmc cells was more stable than DR4 carrying Tn and STn antigens [37, 38]. The presented results indicated that core 1-derived O-glycans (m/z: 955.4, 1316.6) appeared in Jurkat-T-Cosmc and LS174T-Tn^+^-Cosmc cells, rather than in Jurkat T and LS174T-Tn^+^ cells. Although there has been no identification of levels of core 1-derived O-glycans in DR4 or DR5, this does not deny that ST antigen corrects the structures of DR4 and DR5.

Except for core 1- and core 2-derived O-glycans, core 3-derived O-glycan (m/z: 996.4) was only present in LS174T-Tn^−^ cells. Core 3-derived O-glycans are a major type of O-glycans and primarily expressed in the colon, which comprises part of the intestinal mucosal barrier. C3GnT-deficient mice have been shown to be highly susceptible to experimental models of colitis and colorectal adenocarcinoma [39]. Interestingly, compared with Jurkat T, LS174T-Tn^+^, Jurkat T-Cosmc, and LS174T-Tn^+^-Cosmc cells, LS174T-Tn^−^ cells displayed the lowest ability to proliferate and migrate and showed strong apoptosis ability. This suggests that the malignant activity of tumor cells may be controlled by the complexity of O-glycans, despite the fact that the role of core 3-derived O-glycans in malignant behavior of tumor cells is still poorly understood. Since the activity of C3GnT showed no significant differences before and after transfection with WtCosmc in Jurkat T and LS174T-Tn^−^ cells, it was concluded that Cosmc transfection does not affect C3GnT activity and core 3 O-glycan extension. The low C3GnT activity in LS174T-Tn^+^ and LS174T-Tn^+^-Cosmc cells indicated that they were unable to form core 3-derived O-glycans. To be sure, compared to traditional experiments, the novel fluorescence method used for C3GnT activity detection is simpler and faster and prevents contact with radioactive substances. It may serve as a highly suitable method to explore the activity of C3GnT.

In summary, this study clarified that WtCosmc plasmid transfection significantly inhibits the malignant behavior of tumor cells and enhances Apo2L/TRAIL-induced apoptosis by correcting abnormal O-glycosylation. In turn, this changed the structure of DRs on the cell surface and contributed to DR efficiency binding with Apo2L/TRAIL. These preliminary results confirm that the extension of O-glycans on the cell membrane surface by modification of Cosmc cell surface O-glycosylation not only provides new targets for tumor treatment but also provides a novel experimental and theoretical basis for rational clinical treatment of tumors.

## MATERIALS AND METHODS

### Cell lines and cell sorting

Human colon cancer cell line LS174T (Procell CL-0145) and Jurkat T cells (Clone E6-1, Procell CL-0129) were kindly provided by Procell Life Science and Technology Co., Ltd. (Wuhan, China). The cells were cultured in the high-glucose Dulbecco’s modified Eagle’s medium (DMEM), RPMI-1640 medium (Hyclone) plus 10% fetal bovine serum (FBS, Gibco, Grand Island, NY, USA), and 100 U/mL penicillin-streptomycin (Solarbio, Beijing, China) at 37°C and 5% CO_2_. LS174T-Tn^+^ and Tn^−^ cells were separated from LS174T cells using immune magnetic beads according to the literature [40].

Primary mouse anti-Tn mAb (IgM, provided by Dr. Tongzhong Ju at Emory University School of Medicine in Atlanta, GA, USA) was used. Finally, anti-mouse IgM MicroBeads (130-047-301) were purchased from Miltenyi (Bergisch Gladbach, Germany). Sorted cells were continuously cultured and used for subsequent experiments.

### Cosmc transfection

The vectors of GV367-EGFP-Cosmc (WtCosmc, C1GalT1C1 [16164-4]: GAGGATCCCCGG-GTACCGGTCGCCACCATGCTTTCTGAAAGCAGCTCC) and Mock were constructed by Genechem Co., Ltd. (Shanghai, China). Tn^+^ cells (1 × 10^5^) were transfected with WtCosmc (5 × 10^8^ TU/mL) and Mock (5 × 10^8^ TU/mL) according to the manufacturer’s instructions. Cells were transfected with green fluorescent protein and isolated after purification with 5 μg/mL puromycin (Sigma-Aldrich) for 7 days. Then, Tn antigen was detected using flow cytometry (BD Biosciences, NJ, USA).

### Flow cytometry and Tn expression

Cells (5 × 10^5^) were resuspended in 100 μL of phosphate-buffered saline (PBS) and incubated with mouse anti-Tn mAb (IgM) at 4°C for 1 hour (h). Then, cells were incubated with APC-labelled goat anti-mouse IgM (Santa Cruz, CA, USA) for 0.5 h. Finally, cells were analyzed by flow cytometry after washing twice with PBS.

### Western blotting

Cells were lysed in RIPA lysis buffer containing 1 mM phenylmethylsulfonyl fluoride (PMSF, Solarbio Co., Ltd. Beijing, China) at 4°C for 30 minutes (min), then centrifuged at 16,000 × g for 15 min at 4°C to obtain the whole cell extract. Protein concentration in the extract was determined with a bicinchoninic acid (BCA) assay kit (Thermo Fisher Scientific, Waltham, MA, USA). Equal quantities of denatured protein were loaded and separated by 10% sodium dodecyl sulfate polyacrylamide gel electrophoresis (SDS-PAGE, Beyotime, Shanghai, China) and transferred onto polyvinylidene fluoride (PVDF) membranes (Pall, NY, USA), then probed with primary antibodies (Rabbit anti-Human Cosmc: Proteintech, Rosemont, IL, USA, 19254-1-AP, 1:2000) and horseradish peroxidase (HRP)-conjugated secondary antibodies. Further incubation was performed using an enhanced chemiluminescence (ECL) kit (Thermo Fisher Scientific) and exposed after rinsing with TBST (T:Tris; B:Buffer; S:Solution; T:Tween). The protein bands were visualized using a chemiluminescence detection system (ChemisCope, UK). Finally, α-Tubulin was used as a protein loading control.

### Cell proliferation assay

The proliferation of Jurkat T cells was detected using a CCK-8 assay. Cells (3 × l0^4^/well) were seeded in 96-well plates and cultured for 15 h, 30 h, 45 h, and 60 h. Cells were then added to wells (10 μL of CCK-8 reagent per well [Biosharp, Hefei, China]) and continuously cultured for 4 h. The OD_450_ value was measured using a microplate reader (Tecan, M200 Pro, Switzerland).

Proliferation of LS174T cells (LS174T-Tn^−^, LS174T-Tn^+^, LS174T-Tn^+^-Mock, and LS174T-Tn^+^-Cosmc) was observed at 5 min intervals continuously from 0 h to 60 h using a real-time cell DP Analyzer (RTCA, xCELLigence, ACEA Biosciences, CA, USA). Cells (2 × l0^4^/well) in 100 μL of DMEM medium supplemented with 10% FBS and 1% penicillin-streptomycin were seeded in the E-plate and incubated at 37°C and 5% CO_2_. Cell index (CI) was recorded, which reflects changes in cell number at each timepoint. The rate of cell growth was calculated based on the slope of the line between two given timepoints.

### Cell migration assay

Migration of Jurkat T cells was detected using a transwell assay. Cells (2 × 10^4^/well) were seeded into the 8-μm pore transwell chamber (Corning, NY, USA) and cultured for 24 h. Cells that migrated outside the transwell membrane were collected and washed twice with PBS (1200 rpm, 4°C, 10 min). Cell suspensions were replaced in 96-well plates, added to 50 uL of CellTiter-Gloreagent (Promega, WI, USA), and incubated at room temperature for 10 min. The luminescence from each well was recorded by a microplate reader. The migration of LS174T cells was analyzed using a RTCA. The lower chamber of the CIM plates received DMEM supplemented with 10% FBS and 1% penicillin-streptomycin (165 μL/well), while the upper chamber received DMEM without FBS (30 μL/well). Then, cell suspensions (3 × 10^4^/well in 100 μL of DMEM without FBS) were added into the upper chamber and incubated at 37°C and 5% CO_2_. The cell index (CI) values were recorded at 5 min intervals over a 24 h period and used to analyze the dynamic migration of cells in real-time.

### Apo2L/TRAIL-induced cell apoptosis

For cell apoptosis induced by Apo2L/TRAIL, cells were either left untreated or treated with different doses of Apo2L/TRAIL (SinoBio Inc., Beijing, China). The doses of Jurkat T cells were as follows: 10 ng/mL, 20 ng/mL, and 30 ng/mL. For LS174T cells, doses were as follows: 10 ng/mL, 50 ng/mL, and 100 ng/mL. Cells were cultured for 12 h or treated with the same dosage of Apo2L/TRAIL (dose for Jurkat T cells = 20 ng/mL, dose for LS174T cells = 100 ng/mL) and cultured for different durations of time (6 h, 12 h, and 18 h). Apoptosis was evaluated by annexin V-APC/PI (Thermo Fisher Scientific, Waltham, MA, USA) staining according to the manufacturer’s instructions. Cells were resuspended in 1× binding buffer at 5 × 10^6^/mL, then 5 μL of annexin V and 5 μL of propidium iodide (PI) were added. Finally, cells were incubated for 15 min at room temperature. Apoptosis was analyzed using a flow cytometer (BD Biosciences, NJ, USA).

### Cytoplasmic protein extraction and T-synthase activity assay

Cytosolic proteins were extracted from approximately 1 × 10^7^ cells using nuclear and cytoplasmic extraction reagents (Pierce, Dallas, TX, USA) according to the instructions. Protein levels were quantified using a BCA protein assay kit (Pierce). T-synthase activity was measured using a fluorescent assay as described previously [41].

### Core 3 β1-3 N-acetylglucosaminyltransferase (C3GnT) activity assay

During the synthesis process of core 3-derived O-glycans, C3GnT serves as a key enzyme. However, current methods using radioactive substrates to assay its activity are more complex. Therefore, a novel method was developed to detect the activity of C3GnT according to T-synthase activity. This is due to C3GnT utilizing GalNAc-α-4-(MU) as its acceptor substrate and UDP-GlcNAc (Sigma) as a donor to form GlcNAcβ1-3GalNAc-α-(4-MU). The reaction product is cleaved by O-glycosidase to release free 4-MU with high fluorescence. The fluorescence intensity of 4-MU represents the activity of C3GnT product. The experimental procedure is similar to that of detecting T-synthase activity [41]. The procedure is performed as follows.

A total volume of 50 μL of master mixes containing 1 mM GalNAc-α-4-methylumbelliferone (GalNAc-α-4-MU, Santa Cruz), 0.5 mM UDP-Gal (Calbiochem, Darmstadt, Germany), 0.1% Triton X-100 (Solarbio), 20 mM MnCl_2_ (Beilian, Tianjin, China), 800 units of O-glycosidase (New England Biolabs, Ipswich, MA, USA), and 50 mM MES-NaOH buffer (pH = 6.8, Sigma, Covington, LA, USA) was placed in a 96-well black plate. Then, 50 μg of cytoplasmic protein was added to each well and incubated at 37°C for 1 h. The fluorescence intensity was assayed using a fluorimeter (Ex (Excitation Wavelength) = 255 nm/Em (Emission Wavelength) = 460 nm) after 100 μL of Glycine-NaOH (pH = 10.0, Solarbio) was added to stop the reaction. The fluorescence intensity of 4-MU represents the activity of C3GnT. A standard curve was prepared with 4-MU [1 pmol = 50 relative fluorescence units (RFUs)], and C3GnT activity was calculated based on fluorescence intensity [41].

### Profiles of O-glycan as detected by CORA

As previously described [4], CORA was used to analyze the profiles of O-glycans. Approximately 3 × 10^5^ cells were incubated for 3 days with the compound Bn-α-GalNAc (Dr. Tongzhong Ju, Emory University, Atlanta, GA, USA) before collection of media. Bn-O-glycans were purified and separated from flowthrough using a Sep-Pak C18 3 cc cartridge (Waters, Milford, MA, USA) after centrifugation. The O-glycans were eluted with organic solvents, freeze-dried, and permethylated. Extracted polysaccharides were analyzed using matrix-assisted laser desorption/ionization time-of-flight (MALDI-TOF) mass spectrometry (Agilent, Santa Clara, CA, USA).

### Statistical analyses

All experiments were repeated three times. Data were expressed as mean ± standard deviation (mean ± SD). Student’s *t*-tests were used to compare the significant differences between experimental groups. The statistical analysis was performed using GraphPad Prism 8.0 and SPSS 22.0 software. Categorical data were analyzed using the chi-square test. Statistical significance was defined as *P* < 0.05.
